# A Technical, Economic, and Environmental Performance of Grid-Connected Hybrid (Photovoltaic-Wind) Power System in Algeria

**DOI:** 10.1155/2013/123160

**Published:** 2013-12-31

**Authors:** Djohra Saheb-Koussa, Mustapha Koussa, Nourredine Said

**Affiliations:** Centre de Développement des Énergies Renouvelables, BP62 Route de l'Observatoire Bouzareah, 16340 Alger, Algeria

## Abstract

This paper studies the technical, economic, and environmental analysis of wind and photovoltaic power systems connected to a conventional grid. The main interest in such systems is on-site consumption of the produced energy, system hybridization, pooling of resources, and contribution to the environment protection. To ensure a better management of system energy, models have been used for determining the power that the constituting subsystems can deliver under specific weather conditions. Simulation is performed using MATLAB-SIMULINK. While, the economic and environmental study is performed using HOMER software. From an economic point of view, this allows to compare the financial constraints on each part of the system for the case of Adrar site which is located to the northern part of the south of Algeria. It also permits to optimally size and select the system presenting the best features on the basis of two parameters, that is, cost and effectiveness. From an environmental point of view, this study allows highlighting the role of renewable energy in reducing gas emissions related to greenhouse effects. In addition, through a set of sensitivity analysis, it is found that the wind speed has more effects on the environmental and economic performances of grid-connected hybrid (photovoltaic-wind) power systems.

## 1. Introduction

Recently, a growing number of organizations have begun to consider renewable energy and industries related to their production, distribution, and services as opportunities to take rather than regulations [1–6]. Several factors including Kyoto Protocol, alarming reports from the Intergovernmental Panel on Climate Change (IPCC), and Copenhagen climate change conference (COP15) have contributed to this change in opinion, and many countries believe that the trend will continue such that it is very important for them to immediately prepare for the ‘‘green race.” The Algerian government and companies are not exceptions indeed; they have recently intensified their efforts towards promoting the economic growth via supporting green industries. Examples showing such efforts include plans for establishing numerous energy clusters in many areas like the proposed wind farm (project) with a capacity of 10 megawatts launched in Adrar and the hybrid plant in the region of Hassi R'Mel (Laghouat) built and made operational in February 2011. Similar projects, environmentally friendly and producing clean and renewable energy, are scheduled for the areas of Timimoun and Kunta Zaouiet encouraging the use of renewable energy [7, 8].

Electrical energy generation from wind and PV sources is considered as being the most promising renewable energy and is, therefore, to be developed to replace coal, oil, gas, and even nuclear based production. However, any process of transforming energy from one form into another usable form is complex and naturally includes a certain number of economic and environmental features of different kinds (operation of large-scale renewable energy requires space where the resource is available that requires a “good” management planning and the electrical networks will also be adapted and managed so as to promote decentralized production). The obtained technoeconomic and environmental results allow reaching an objective judgment regarding the studied system.

## 2. Presentation of the Different Systems of Distributed Generation from Renewable Energy 

*Standalone Systems*. For standalone installations, the energy produced employing photovoltaic solar panels or wind generators is either immediately consumed (pumping, ventilation, lighting, refrigerator, etc.) or stored in batteries for later use. The produced current is either directly fed to the consuming equipment or converted using an inverter to supply devices that require AC power [10, 11].

*Multisource Hybrid Systems*. These systems supply electricity that is often used at remote sites and are built coupling different sources of production of electrical energy such as wind, solar, and others. They also allow a more reliable supply of electricity. Nearly two billion people are not connected to the utility grid (44% of the world population). Thus, the development of hybrid systems for renewable energy conversion will undoubtedly help to solve many social problems, especially in poor countries, and open up vast commercial markets [10, 12–15].

**Figure 1:**
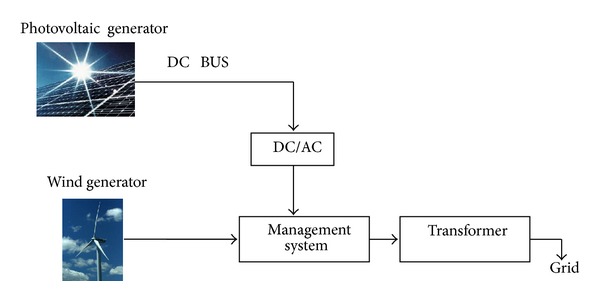
Decentralized installation connected to the grid.

*Distributed Systems Connected to the Grid*. These are usually medium and large systems which are grid connected [16–19] and which, in general, produce electrical energy amounts depending on sunshine and wind conditions (Figure 1).

## 3. Characteristics of the PV-Wind Systems Considered

The architecture presented in Figure 1 is the one upon which this paper is based. With this type of PV-wind system for the generation of electrical energy the objective is to inject into the grid the energy thus generated.

For the design, the power production of the different sources becomes freely controllable without affecting the state values of the grid. Decoupling the state values means that the variations of the renewable resources like the velocity of the wind and the intensity of the solar radiation will not influence the state values of the electrical grid as the harmonics generation, flickers, frequency fluctuation, under voltage, and over voltage. These values are only controlled by the inverter for the photovoltaic generator and by the microprocessor-controlled OptiTip pitch regulation, ensuring continuous and optimal adjustment of the angles of the blades in relation to the prevailing wind, for the wind generator. On the other hand, changes in the loads, which influence the state values of the grid, will not affect the generation side.

A control/management strategy is developed for this architecture to operate it in the highest efficient way. The efficiency here means the most utilization of the renewable energy sources in order to minimize the cost of the produced energy while preserving the reliability of the system.

## 4. Methodology

### 4.1. Theoretical Aspect of the Modeling

#### 4.1.1. Modeling of the PV System

For each hour h in a year, the power delivered by a PV generator *P*_pv-h_ (W) is described by the IV characteristic which varies with the hourly solar radiation *G*_h_ and the hourly dry temperature *T*_h_. This is given, in algebraic form, by

(1) $$\begin{array}{c} I_{\text{pv-h}}=f\left(V_{\text{ph-h}},G_{\text{h}},T_{\text{h}}\right). \end{array}$$

Singer model [20, 21] has been used and MATLAB-SIMULINK program was developed for this purpose.

*MPPT Algorithm*. In MPPT (maximum power point tracking) operation, the PV array produces maximum power under variable conditions of solar radiation and ambient temperature. The MPPT algorithm which is used in this work is the incremental conductance algorithm (IC). Conductance algorithm is based on the differentiation of PV power and on condition of zero slope of PV curve. The MPPT can be tracked by comparing the incremental conductance *dI*_pv-h_/*dV*_pv-h_ to the instantaneous conductance /*V*. Therefore the sign of the quantity *dI*_pv-h_/*dV*_pv-h_ + *I*_pv-h_/*V*_pv-h_ indicates the correct direction of perturbation leading to the MPPT. When MPPT has been reached, the operation of the PV is held at this point and perturbation is stopped. If a change in *dI* is presented, the algorithm increments or decrements the *V* to track the new MPPT the increment (or decrement) size determines how fast the MPPT is tracked.

When the optimum operation point of PV curve is to the left of the MPPT, we have *dI*_pv-h_/*dV*_pv-h_ + *I*_pv-h_/*V*_pv-h_≺0 thus a reduction in PV's voltage is essential to achieve MPPT.

Similarly, when the optimum point is to the left of the MPPT, we have *dI*_pv-h_/*dV*_pv-h_ + *I*_pv-h_/*V*_pv-h_≻0; thus an increase in PV's voltage is essential to achieve MPPT. Traditionally these changes in PV's voltage may be done by coupling a DC/DC converter to PV and controlling properly its duty cycle. In the present study the used DC/DC converter in MPPT is the boost due to easy way of duty cycle control [22].

**Figure 2:**
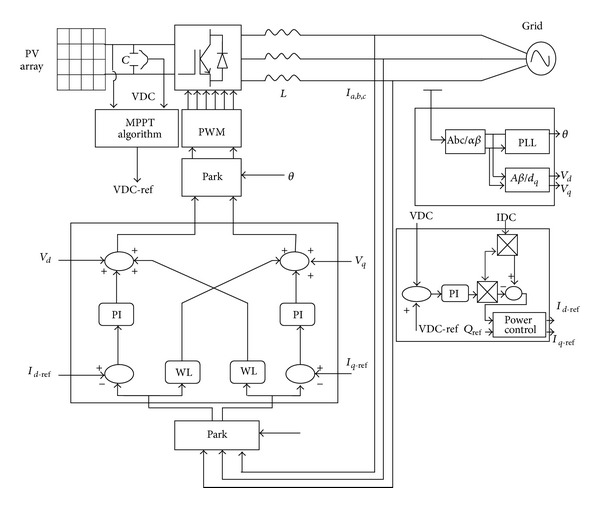
Schematic diagram of a connected PV-grid [9].

*Inverter*. In this paper the connection of PV system to the grid takes place in one stage using a voltage source inverter. In Figure 2 we can see that between the PV generator and the inverter only one capacitor exists. Based on the IC algorithm, when the output voltage of PV generator is changed, the MPPT changes simultaneously. For the implementation of IC algorithm directly to the inverter, the switching elements of the inverter must be appropriately pulsed so that every moment the voltage capacitor of the DC bus is equal to the reference voltage which is given by MPPT algorithm (VDC-ref). Therefore, the algorithm brings in the capacitor voltage and the PV's current as inputs and the desirable PV's voltage (which is capacitors new reference voltage) as output [22].

**Figure 3:**
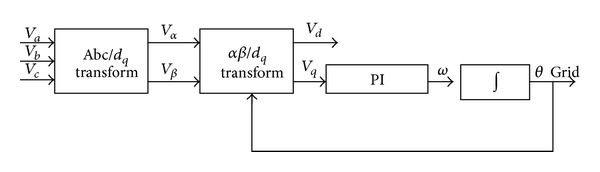
Transport delay-based PLL algorithm.

*Control and Synchronization with the Grid*. In all the power conversion chains, it should be noticed that the inverter output voltages must be synchronized with the distribution grid [23]. For that purpose, we introduced and turned a phase locked loop (PLL) which delivers the angle *θ* = *ωt* mandatory for the Park transformation (translation into the synchronous frame).  Figure 3 shows the block diagram of this PLL algorithm. While supplying to the load or the grid a current corresponding to the real power reference, a swing is created between voltages and currents in order to deliver a reactive power according to the command.

A proportional integral controller is used to control the active and reactive power flowing.

#### 4.1.2. Modeling of the Wind System

*Wind Speed Variation with Height [24]*. To calculate the output of the wind turbine in each of the 8760 hours in a year, the hourly values of measured wind speed in the Adrar site at the hub height of the machine is calculated by using

(2) $$\begin{array}{c} v_{\text{hub-h}}=v\frac{\ln\left(z_{\text{hub}}/z_{0}^{}\right)}{\ln\left(z_{\text{data}}/z_{0}\right)}, \end{array}$$

where *z*_hub_ is the hub height of the wind turbine (m), *z*_data_ is the anemometer height (m), *z*_0_ is the surface roughness length (m) [25], and the *v*_hub-h_ is the wind speed at the anemometer height during the hour h (m/s).

*Dynamic Model*. The types of wind turbine generators may vary from wind generator to wind generator due to different generator types (asynchronous, synchronous), especially the circuitry connecting the wind generator to the three-phase grid which can have two different forms (direct or indirect grid connection).

**Figure 4:**
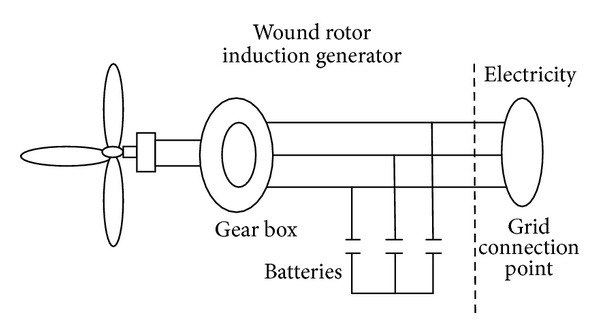
Wind system block diagram.

In the studied case, the generator has been connected directly to the three-phase alternating current grid. The resulting configuration is simple and, according to the literature [26, 27], is widely used in practice (Figure 4).

*Wind Turbine Dynamics Model*. The mechanical power extracted from the wind [28, 29] is given by the following relation:

(3) $$\begin{array}{c} P_{t}=\frac{1}{2}\rho^{}{C_{p}}_{}{\left(\lambda_{},\beta \right)}^{}S_{}v^{3}, \end{array}$$

with

(4) $$\begin{array}{c} S=\pi_{}R_{t}^{2}, \end{array}$$

where *S* is the area swept by the wind in m²; *ρ* is the air density equal at standard conditions 1.294 kg m^−3^; *v* is the wind speed in m/s; *R*_*t*_ is the radius in m.

The power coefficient *C*_*p*_ depends on the tip speed ratio (*λ*) and the blade angle (*β*). In the case of turbines without pitch control, the blade angle is constant and *C*_*p*_ values only depend on those of *λ*, the tip speed ratio being expressed as follows:

(5) $$\begin{array}{c} \lambda =\frac{\Omega_{t}R_{t}}{v}. \end{array}$$

Also, the mechanical torque produced by the wind is expressed in the following relation:

(6) $$\begin{array}{c} T_{t}=\frac{P_{t}}{\Omega_{t}}. \end{array}$$

The VESTAS 47-660 wind generator experimental power output data given by the manufacturer as well as the *C*_*p*_ curve [30] have been approximated by using a simple MATLAB polynomial interpolation, available as polyfit polynomial function and the obtained correlations are expressed as follows:

(7) P(v)=4240−4727v−2194v2−562v3+88.5v4−8.91v5+0.585v6−0.0249v7+6.64·10−4v8Cp(v)=1.1072−1.2698v−0.4931v2−0.00084v3+0.0781v4−4.27·10−4v5+1.37·10−5v6−2.44·10−7v7+1.83·10−9v8.

Then, the operation of the wind turbine system is simulated using the wind turbine equations which evaluate the mechanical torque (*T*_*t*_) and requires the turbine angular speed (*Ω*_*t*_) and the wind speed (*v*) as data.

*Drive Drain Dynamics Model*. To evaluate the generator speed (*Ω*_am_), the generator torque (*T*_am_) and the turbine torque (*T*_*t*_′) are required as data. The dynamic behaviour of the mechanical system is determined by using the classical rotational dynamics equations. The inertia is considered as concentrated in one lumped mass including the contribution of blades, generator shafts, and gear box. The dynamic motion equation of the mechanical system has the following expression:

(8) $$\begin{array}{c} J\frac{d^{}\Omega_{\text{am}}}{dt}+{f_{v}}_{}\Omega_{\text{am}}=T_{t}^{\prime }-T_{\text{am}}. \end{array}$$

The turbine torque and generator speed (*T*_*t*_′ and *Ω*_am_) and the mechanical torque and turbine speed (*T*_*t*_ and *Ω*_*t*_) supplied to the wind turbine block simulation have been linked by means of the gear box whose ratio is *k* (*k* = 50.5) for the VESTAS 660-47 wind generator.

*Asynchronous Machine Dynamics Model.* The asynchronous machine equations permit to calculate the electrical power generation of the system and return the generator torque *T*_am_, the active and reactive power. The generator speed *Ω*_as_ and voltage *V*_*d*,*q*_ are the required input parameters, obtained by using the Park transform block PT of the three-phase system into the two-phase system [31].

*(a) Electric and Magnetic Equations.* The voltage equations representing an induction machine [29, 32] in an arbitrary reference frame can be written, in terms of the phase currents, as given by [32].

In the present model, the stator and rotor voltages along the *d* and *q* axes are given by [32].

Using these last ones, a model for wound rotor generators can be developed. This latter has short circuited windings and, as a consequence, rotor voltages evaluate to zero (*V*_*dr*_ = 0 and *V*_*qr*_ = 0) in this case. Therefore, the representative wound rotor generator equations are

(9) $$\begin{array}{c} s\varphi_{ds}=V_{ds}-R_{s}I_{ds}+\omega_{s}\cdot \varphi_{qs}\\ s\varphi_{qs}=V_{qs}-R_{s}\cdot I_{qs}-\omega_{s}\cdot \varphi_{ds}\\ s\varphi_{dr}=-R_{r}\cdot I_{dr}+\left(\omega_{s}-\omega_{r}\right)\cdot \varphi_{qr}\\ s\varphi_{qr}=-R_{r}\cdot I_{qr}-{\left(\omega_{s}-\omega_{r}^{}\right)}_{sr}\cdot \varphi_{dr}, \end{array}$$

where

(10) $$\begin{array}{c} \varphi_{ds}=L_{s}\cdot I_{ds}+L_{sr}\cdot I_{dr}\\ \varphi_{qs}=L_{s}\cdot I_{qs}+L_{sr}\cdot I_{qr}\\ \varphi_{dr}=L_{r}\cdot I_{dr}+L_{sr}\cdot I_{ds}\\ \varphi_{qr}=L_{r}\cdot I_{qr}+L_{sr}\cdot I_{qs}. \end{array}$$

*(b) Evaluation of the Electromagnetic Torque*. The electromagnetic torque has been calculated by employing (11), proposed by [29, 32].

Consider

(11) $$\begin{array}{c} T_{\text{am}}=\left(\frac{3}{2}\right)P\cdot L_{sr}\left(I_{sq}\cdot I_{rq}-I_{sd}\cdot I_{rq}\right). \end{array}$$

*(c) Evaluation of Real and Reactive Power.* The active (*P*) and reactive (*Q*) power have been calculated by using the following equations:

(12) $$\begin{array}{c} \left\{\begin{array}{c} P=V_{ds}I_{ds}+V_{qs}I_{qs}\\ Q=V_{qs}I_{ds}^{}-V_{ds}I_{qs} \end{array}\right\} \end{array}$$

(13) $$\begin{array}{c} \left\{\begin{array}{c} P_{r}=-\left(V_{dr}I_{dr}+V_{qr}I_{qr}\right)\\ Q_{r}=-\left(V_{qr}I_{dr}^{}+V_{dr}I_{qr}\right) \end{array}\right\}. \end{array}$$

Using these equations, a model for wound rotor generators can be developed. This type of generator has short circuited windings; thus the corresponding rotor voltages go to zero (*V*_*dr*_ = 0, *V*_*qr*_ = 0). Taking into account these conditions, (13) give *P*_*r*_ = 0 and *Q*_*r*_ = 0.

## 5. Simulation

The energy system components are photovoltaic modules, wind turbine, grid, and power converter. This study develops a suitable assembly of the key parameters such as photovoltaic array power, wind turbine power curve, battery storage, and converter capacity to match the predefined load. For economic analysis, the cost including the initial capital, replacement cost, and operating and maintenance cost are considered as simulating conditions.

*Photovoltaic Arrays.* The initial cost of photovoltaic arrays may vary from $4.00 to $5.00 per watt. Considering a more optimistic system, the costs of installation, replacement, and maintenance of a 1 kW solar energy system are taken as $5000 and $4000. Sizes of the photovoltaic arrays are varied between 0, 100, 200, 300, 400, 500, 600, and 700 kW.

*Wind Turbine*. Energy generation form wind turbine depends on wind speed variations. The wind turbine rated power should be greater than average electrical load. Therefore, according to the load data discussed above, the average load is around a 7.7 MW. Therefore, a VESTAS 47–660 turbine manufactured by VESTAS wind power is used. Its rated power is 660 kW AC.

*Grid*. Grid exists as the main power component in this hybrid renewable energy system. Moreover, grid has the functions as a storage system, so a grid power system does not need a battery.

*Power Converter*. A converter is required for systems in which DC components serve an AD load or vice versa. For a 1 kW system the installation and replacement costs are taken as $800 and $750, respectively. Lifetime of a unit is considered to be 25 years with an efficiency of 90%.

For the considered system, it is necessary to simulate, during all the hours in a complete year, all the possible designs. Variables are considered hourly, and, therefore, there will be 8760 in a year. At the end of the simulation, we will know the quantity of electrical energy from a PV generator injected to the grid in the year and the electrical energy from wind turbines that is also injected [24].

**Figure 5:**
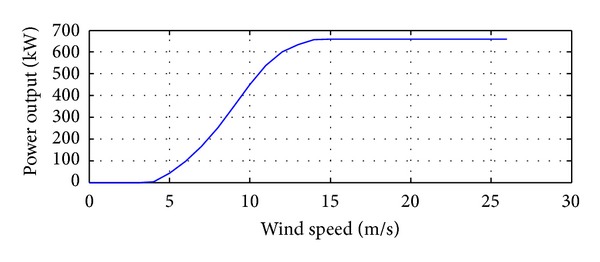
Typical wind turbine power curve.

As a sample site, the Adrar site has been chosen and the following data are used as input:

- the hourly global and diffuse radiation measured on a horizontal plane and the ambient temperature. From the data collected on a horizontal plane, the components of the solar irradiance have been projected onto the surface of a PV panel. Moreover, the inclination of the used solar panel corresponds to the yearly optimum slope as indicated in [33];
- the measured wind speeds and the electromechanical characteristics of a wind turbine, of the VESTAS 47–660 type. This is a three-blade model with a diameter of 46 m, a speed multiplier ratio of 50.5 and a hub height of 100 m [30]. Moreover, the power produced by this wind turbine has been calculated using the power curve (Figure 5), provided by the manufacturer.

### 5.1. Power Produced by the Photovoltaic Generator

For calculating the output characteristics of the photovoltaic system, a program has been developed which requires the global incident radiation and the air temperature as main input data. So, the research unit in renewable energy U.R.E.R. of Adrar provided hourly measured values over a full year of global and diffuse irradiation on a horizontal plane together with those related to the ambient temperature. From the global radiations on the horizontal plane collected data and based on the equations given in [21], the developed program calculates the overall incident irradiations on the surface of the PV panel. These latter and the ambient temperatures are used to calculate the power and current delivered by the PV generator.

**Figure 6:**
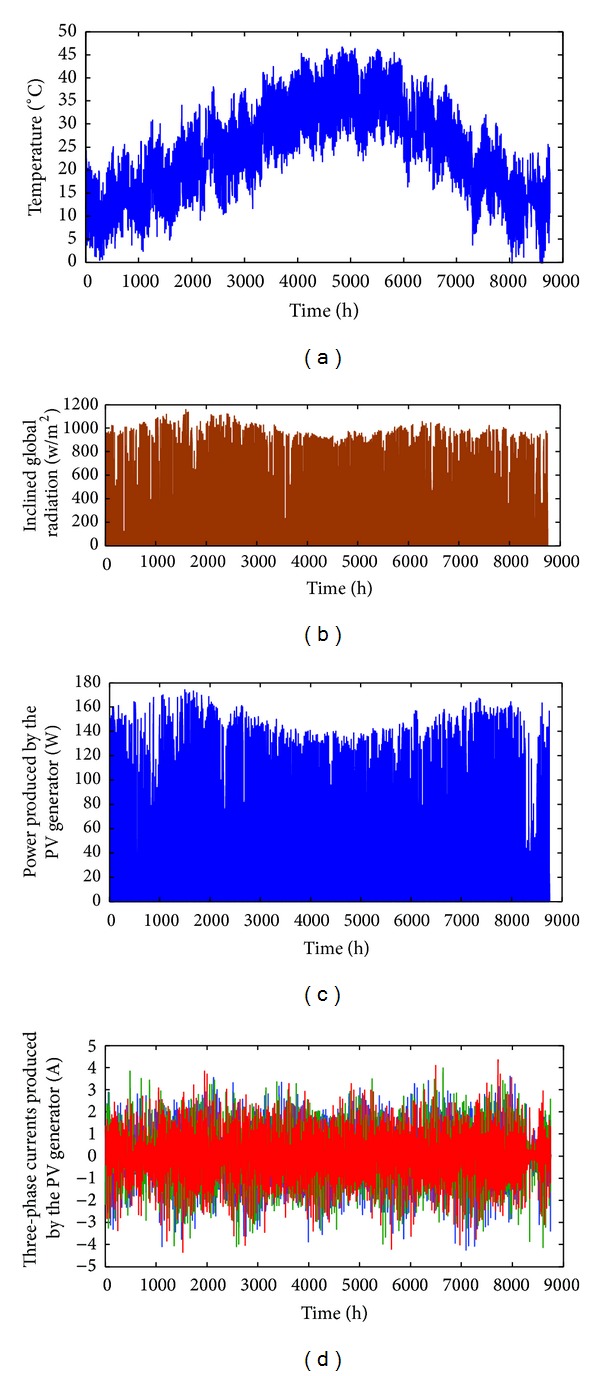
Representation of climatic characteristics, power, and current produced by the photovoltaic module BP SX 150 S installed on the Adrar site.

The obtained results are presented in Figure 6.

### 5.2. Power Produced by the Wind Generator

Generally, to calculate the power generated by a wind turbine, we use the data drawn from the main characteristic *p* = *f*(*v*) related to the turbine and supplied by the manufacturer (Figure 5). In this study, using the equations given in [34], the hourly values of the wind turbine are read from a file in which the wind speed for each hour of the year is given.

**Figure 7:**
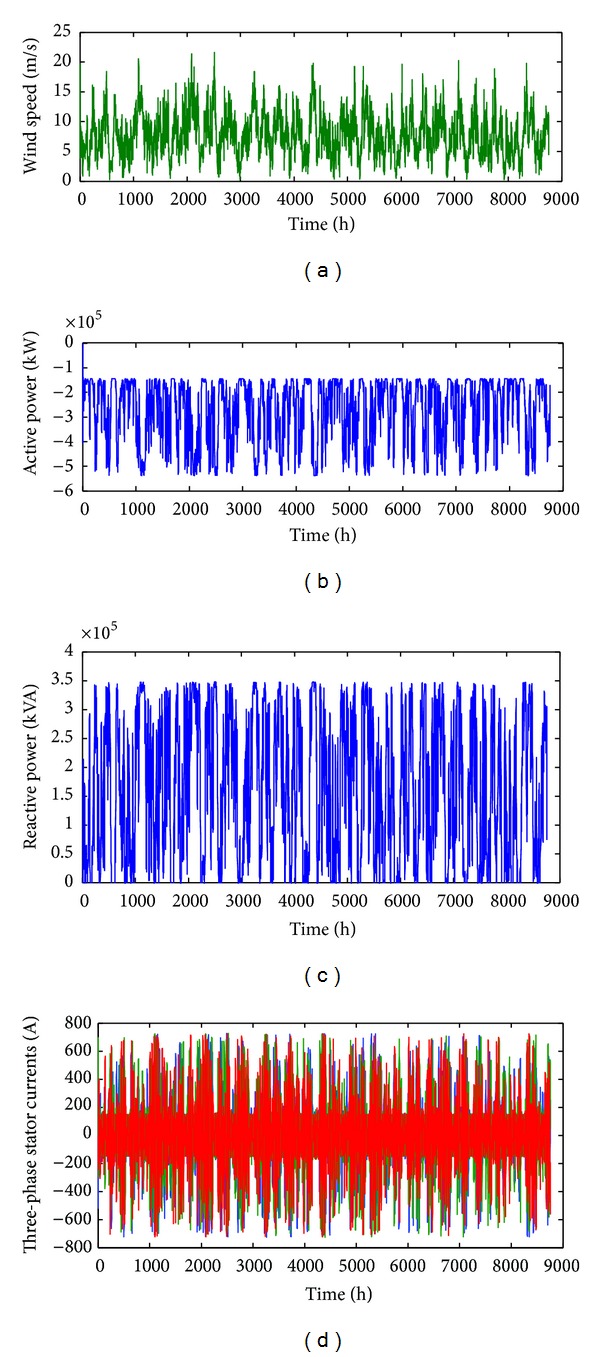
Simulation results.

By using the power curve of the wind turbine, the output power is calculated. With the speed at the hub of the wind turbine, and using its power curve, the power that the wind turbine provides in an hour h, *p*_w-h_ (W) is obtained. If there are *N*_*w*_ wind turbine connected in parallel, this amount is multiplied by *N*_*w*_ [24]. The obtained results are as follows:

- Figure 7(a) shows the evolution of hourly wind speeds;
- Figure 7(b) shows the plots of the active power developed at the asynchronous machine terminals;
- Figure 7(c) shows the plots of the reactive power developed at the asynchronous machine terminals;
- Figure 7(d) shows the current delivered by the wind generator.

### 5.3. Management of the System [24]

**Figure 8:**
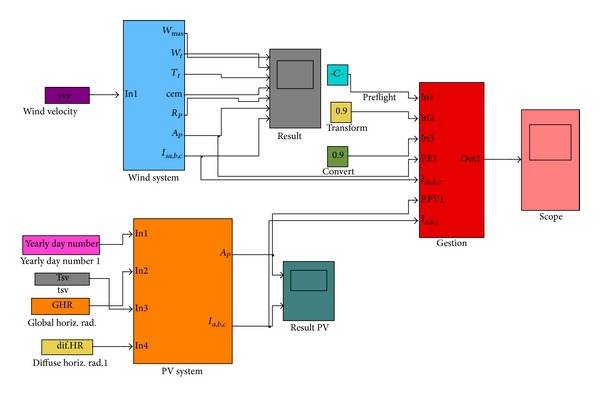
Management of the system.

Figure 8 presents the MATLAB-SIMULINK program diagram of the hybrid system. In this system the power delivered by each of the system devices (PV or wind) should be managed in such a way that the surplus of power produced by any of them is conducted to the grid without giving rise to any phenomena leading to a disturbance of any of these devices. These last ones disturbed by generating harmonics that may distort the grid waveform, flickers, high frequency wave, frequency fluctuation, under voltage, and over voltage.

Thus, for each hour of the year h, the amount of electrical energy available at the transformer connected to the grid is evaluated by [24]

(14) $$\begin{array}{c} P_{\text{AC-h}}={\left(P_{\text{pv-h}}\eta_{\text{INV}}+P_{\text{w-h}}\right)}^{}\eta_{\text{TR}}, \end{array}$$

where *η*_INV_ is the inverter efficiency rate, modeled as a variable depending on the power delivered by the inverter, *η*_TR_ is the efficiency rate of the transformer connected to the electrical grid, including the losses of power in the transmission lines, *P*_w-h_ is the power (W) generated by the wind generator within an hour time, and *P*_pv-h_ is the power (W) generated by the PV generator within an hour time.

However, the amount of power that can be injected each hour into the grid, *P*_EE-h_ (W), cannot be higher than the allowed evacuation capacity at the point of connection to the grid, *P*_MAX-GRID_ (W) [24]:

(15) $$\begin{array}{c} P_{\text{EE-h}}=\min\left(P_{\text{MAX-GRID}},P_{\text{AC-h}}\right), \end{array}$$

Where *P*_MAX-GRID_ (W) is the maximum power evacuation value allowed, which the Algerian law fixes for 20 to 30% of the line thermal limit at the point of connection.

**Figure 9:**
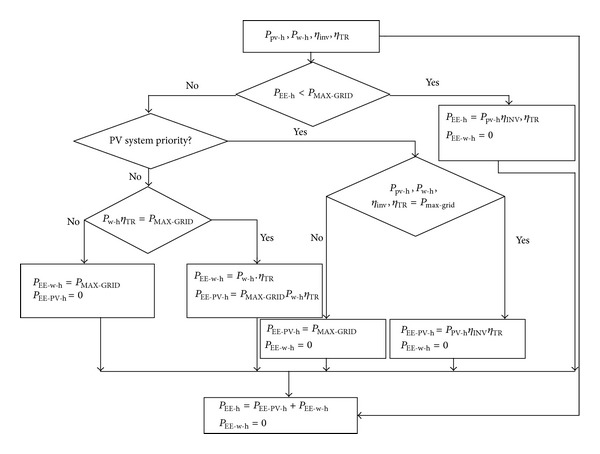
Flowchart for calculating the amount of power to be inject into the grid.

The amount of energy to be injected into the grid obtained from the PV generator (*P*_EE-PV-h_) and the wind generator *P*_EE-w-h_ will be calculated as indicated by Figure 9.

**Figure 10:**
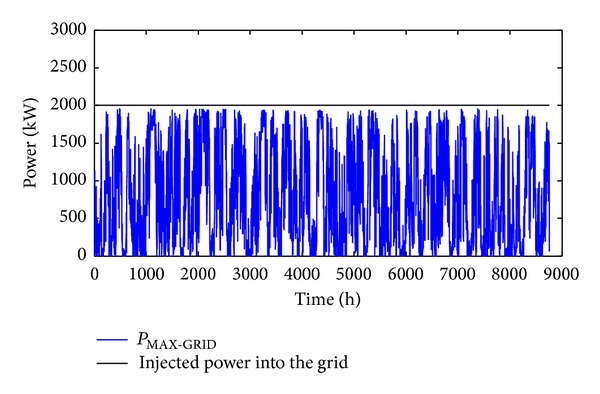
Power injected into the grid.

The results obtained in the case of the previously described scenario are represented in Figure 10 which shows that the hourly produced power injected into the grid is lower than *P*_MAX-GRID_.

### 5.4. Total Annual Production

**Figure 11:**
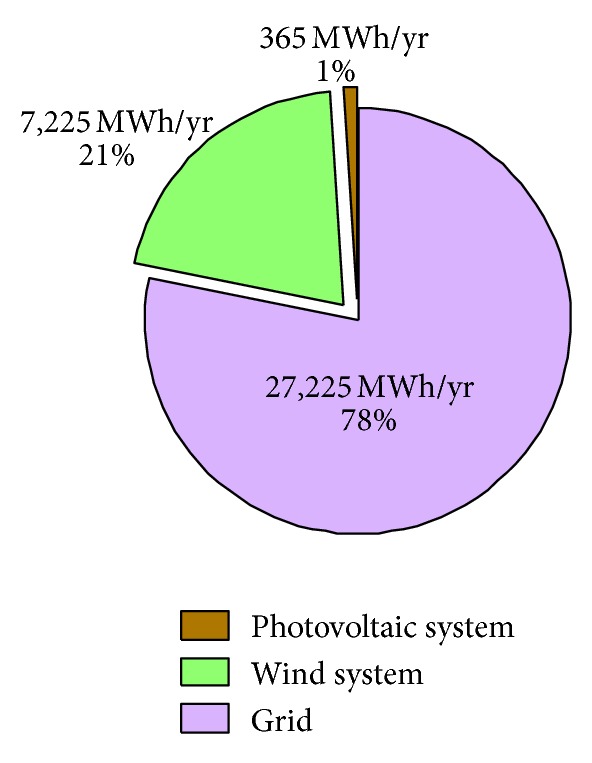
Structure of the production system (PV-wind-grid).

The contribution of each part of the hybrid system (PV-wind-grid) to satisfy a specific load of the 34,815 MWh/yr is shown in Figure 11. It is to be noted that the PV generator produces only 365 MWh/year and covers only 1% of the load. The wind generator, in turn, produces 7,225 MWh/year which constitute nearly 21% of load requirements against a covered load rate of 78% (27,225 MWh/year) provided by the conventional electricity grid [35]. These results are explained by the fact that HOMER software promotes the wind system because of it's efficiency which is very higher than that of PV system.

### 5.5. Hours of Operation

**Figure 12:**
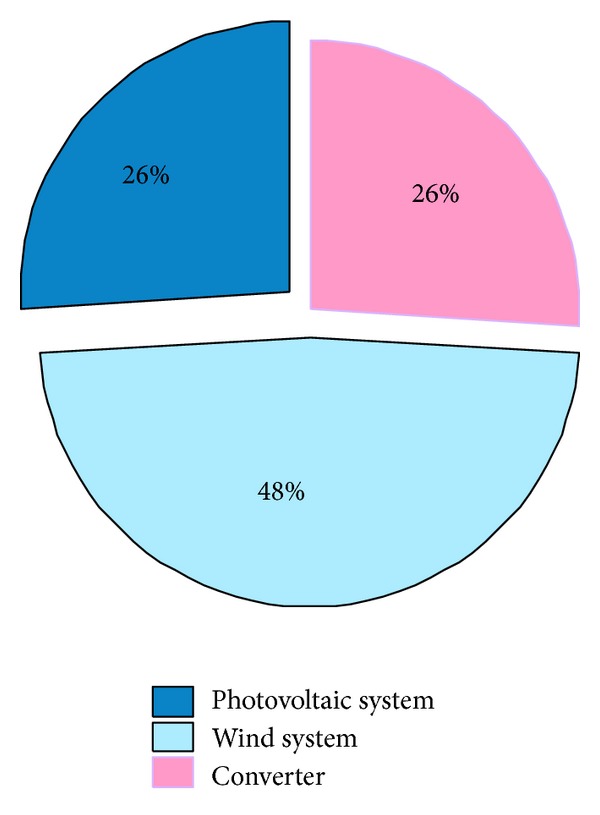
Distribution of operation hours of each of the renewable energy equipment of the hybrid PV-wind-Grid system.

Figure 12 represents the duration of the operation (in hours) of each of the renewable energy equipment of the hybrid PV-wind-Grid system. It is found that the wind generator works over the longest time interval with a 48% rate of the total period, followed by the PV generator and inverter with a rate of 26% for each.

### 5.6. Economic Aspects

The HOMER optimization model [24] uses relatively simple strategies based on the ones studied by Barley et al. [36] and it is able to obtain an optimal design of a hybrid system by selecting the most appropriate strategy.

**Figure 13:**
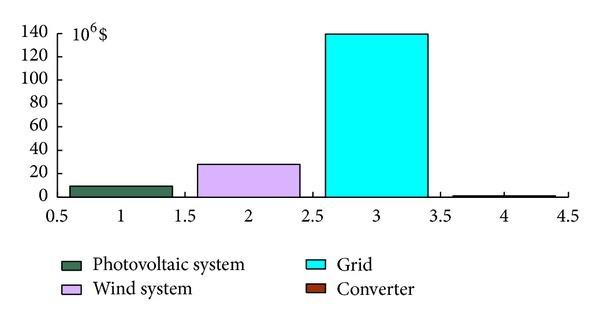
Net present cost.

**Figure 14:**
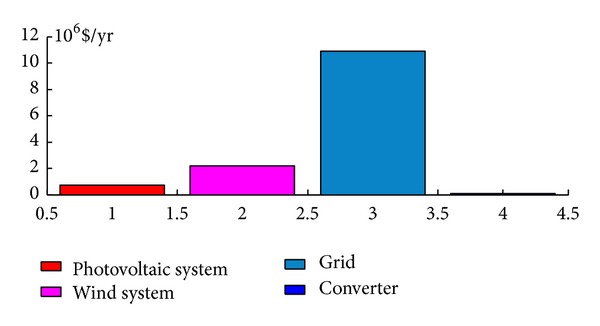
Annual net cost.

Thus, from an economic point of view, it is found that the system composed of a 200 kW rated PV system and three 660 kW rated wind turbines can cover 22% of the electrical energy demand and has a net present cost (NPC) of $177 million and a cost of energy per kilowatt hour (COE) of $0.399/kWh. A comparative economic analysis between the conventional and the optimized system (PV-wind system) employing HOMER software package [35] has been performed and the results are presented in Table 1. From these results, it is noticed that the hybrid system (PV-wind-grid) is more economical than the conventional system if the price per produced kWh of the latter is set to $0.4/kWh. However, the net present cost is calculated for a project lifetime period of 25 years and on the basis of an interest rate of 6%. Figures 13 and 14 show the details of the corresponding costs to each of the systems and the related annual costs [37].

### 5.7. Environmental Aspects

The results regarding the effects of each system configuration, that is, the conventional grid and the hybrid system (PV-wind-grid), on the environment obtained by again using HOMER software in the case of the Adrar site are shown in Table 2 [37]. In this table the quantities of the main gases that are harmful to the environment including CO_2_, SO_2_, and NO_*x*_ are presented. From these results, it is found that the PV-wind-grid hybrid system produces a reduction in the carbon dioxide gas, sulfur dioxide, and nitrogen oxide rates by, respectively, 20%, 22%, and 22% as compared to the quantities produced by the conventional system [37].

### 5.8. Sensitivity Results

In this study, sensitivity analysis was done to study the effects of variation in the solar irradiation and wind speed. The simulation software simulates the long-term implementation of the hybrid system based on their respective search size for the predefined sensitivity values of the components. The emissions, renewable fraction, NPC, and COE are simulated based on the three sensitivity variables: wind speed (m/s), solar irradiation (kW/m^2^/day), and grid electricity price ($/kWh). A long-term simulation for every possible system combination and configuration was done for a one year period (from January 1st 2005 to December 31st 2005). In the present case, solar irradiation is set as sensitivity variables: *G* = 3.5, 4.5, 5, 5.5, 5.72, 7.8, 8 kW/m^2^/day, while wind speed are *v* = 6.9, 7, 7.5, 7.8, 8, 8.8 m/s. Moreover, the grid electricity price is also defined as a sensitivity variable (*p* = 0.1, 0.2, 0.3, 0.4 $/kWh). A total of 192 sensitivity cases were tried for each system configuration. The simulation time was 23 minutes and 46 seconds on a personal computer with Intel CORE Intel Core Duo Processor of 2.53 GHz and a RAM of 2 GB. The sensitivity results in terms of solar irradiation, wind speed, and grid electricity price analyze the feasibility of each system. Here the feasibility of hybrid renewable energy system is analyzed based on emission reduction and cost saving. This type of sensitivity analysis of the systems provides information that a particular system would be optimal at certain sensitivity variables [19]. The PV-wind system is feasible when the grid electricity price is more than $0.4 kW/h. Under this condition, the RF can be between 0.21 and 0.22. A PV-wind system is feasible when global solar irradiation is more than 5.72 kWh/m^2^ per day and the grid electricity price is more expensive than $0.4 kWh. Based on the optimization results, wind energy production shows a bigger proportion of energy generation than solar. While the solar power occupies less than 1% of the total energy generation, wind power occupies approximately a quarter.

**Figure 15:**
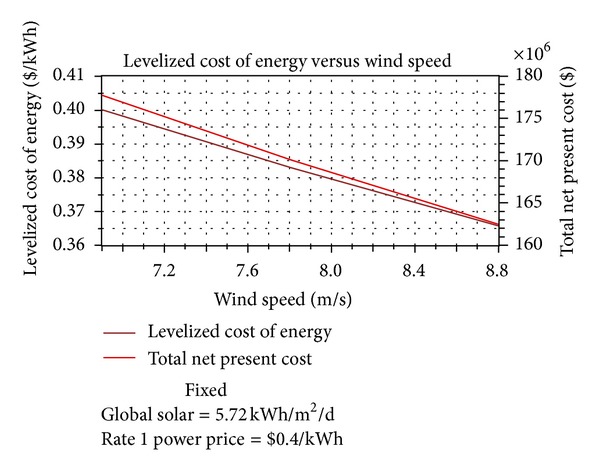
The relationship between cost and wind speed.

**Figure 16:**
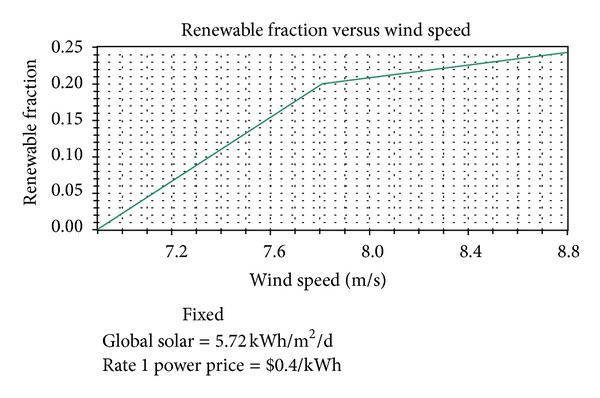
The relationship between RF and wind speed.

**Figure 17:**
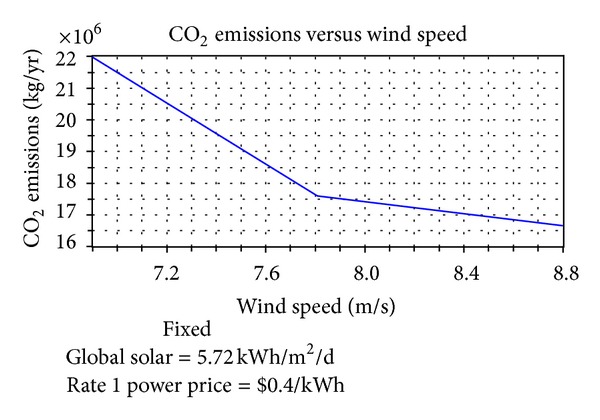
The relationship between CO_2_ emissions and wind speed.

**Figure 18:**
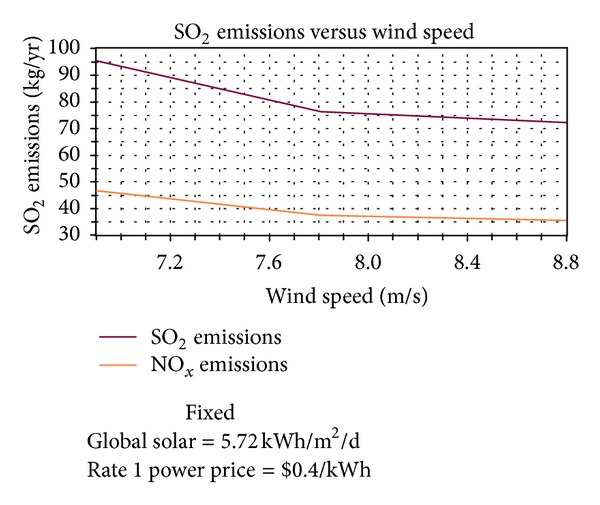
The relationship between sulfur dioxide and nitrogen oxide emissions and wind speed.

Therefore, the wind energy resource has more impact on the implementation. Figures 15–18 reflect the cost, renewable fraction, and emission variation dependent on the sensitivity variable wind speed. The NPC and COE of the hybrid power system reduce when the wind speed increases from 6.9 m/s to 8.8 m/s, Figure 15.

Simultaneously, as seen in Figure 16, renewable fraction rises sharply from 0 to 16% (when wind speed increases from 6.9 to 7 m/s) and then steadily increases to 24% at a slower rate. In addition, as shown in Figures 17 and 18, the main emissions of carbon dioxide, sulfur dioxide, and nitrogen oxide persistently decrease 22%.

## 6. Problems Encountered in Decentralized Systems [37] 

The major difficulty associated with decentralized energy sources is that they generally do not participate in ancillary services (voltage control, frequency, ability to operate in standalone mode, etc.). This is especially true for renewable energy sources whose power flow is unpredictable and very volatile. The integration of decentralized energy generation into power networks raises the following problems:

- random and unpredictable energy production (wind, solar);
- lack of power-frequency control;
- no voltage adjustment performed;
- sensitivity to voltage dips;
- significant sensitivity to changes in primary source (wind, solar) energy levels.

The failure to take part in system services makes this type of sources behave as passive generators, from the electrical energy generation point of view. The penetration of distributed energy generation must be limited from 20 to 30% of the consumed power in order to guarantee acceptable system stability [19].

## 7. Conclusion 

This study is related to the technical, economic, and environmental impact of grid-connected decentralized systems to which an appropriate management of energy is applied and by means of which are developed models of the different parts. The solar and wind energy resource data are collected from the weather station of Adrar which is a typical arid region. The most significant results are as follows.

The power that any subsystem can deliver depends on the weather conditions of the considered site.

The PV based system only covers 1% of the total load consumption. On the other hand, the wind generator contribution amounts to about 21% of the energy production while the remaining 78% are supplied by the conventional electricity grid.

From an economic point of view, it is found that for the Adrar site, which is characterized by a high wind potential, the hybrid system is competitive compared with the conventional system with a cost of the energy COE produced by the network equal to $0.4/kWh since the estimated COE related to the hybrid system is equal to $0,399/kWh, based on an average wind speed greater than or equal to 6 m/s.

From an environmental standpoint, the rates of greenhouse gases (CO_2_, SO_2_, and NO_*x*_) emissions are reduced from 20 to 22% in the case of a hybrid system, compared with the conventional system.

The sensitivity analysis indicates that PV-wind hybrid system is feasible under the meteorological conditions in Adrar region. With the increasing wind speed, the NPC, COE, and emissions of the hybrid renewable energy system reduce, and renewable fraction grows up.

## Figures and Tables

**Table 1:** Cost comparison between a standard system (grid) and a hybrid system (PV-wind-grid).

Cost	Conventional system grid	Hybrid system PV-wind-grid
NPC ($/yr)	177,714,200	177,090,600
COE ($/kWh)	0.4	0.399

**Table 2:** Emissions comparison between a standard system (grid) and a hybrid system (PV-wind-grid).

Pollutant Kg/yr	Conventional system grid	Hybrid system PV-wind-grid
CO_2_	21,965,160	17,199,524
SO_2_	95,229	74,568
NO_X_	46,572	36,467
